# Anti-invasive effects of minoxidil on human breast cancer cells: combination with ranolazine

**DOI:** 10.1007/s10585-022-10166-7

**Published:** 2022-05-28

**Authors:** Shiwen Qiu, Scott P. Fraser, Wayne Pires, Mustafa B. A. Djamgoz

**Affiliations:** 1 Department of Life Sciences, Imperial College London, South Kensington Campus, London, SW7 2AZ UK; 2 Present Address: Department of Pharmacology, Yong Loo Lin School of Medicine, 10 Medical Dr, Singapore, 117597 Singapore; 3 Biotechnology Research Centre, Cyprus International University, Haspolat, Mersin 10, Turkey

**Keywords:** Invasion, Voltage-gated sodium channel, K_ATP_ channel, Minoxidil, Ranolazine, Repurposing

## Abstract

A plethora of ion channels have been shown to be involved systemically in the pathophysiology of cancer and ion channel blockers can produce anti-metastatic effects. However, although ion channels are known to frequently function in concerted action, little is known about possible combined effects of ion channel modulators on metastatic cell behaviour. Here, we investigated functional consequences of pharmacologically modulating ATP-gated potassium (K_ATP_) channel and voltage-gated sodium channel (VGSC) activities individually and in combination. Two triple-negative human breast cancer cell lines were used: MDA-MB-231 and MDA-MB-468, the latter mainly for comparison. Most experiments were carried out on hypoxic cells. Electrophysiological effects were studied by whole-cell patch clamp recording. Minoxidil (a K_ATP_ channel opener) and ranolazine (a blocker of the VGSC persistent current) had no effect on cell viability and proliferation, alone or in combination. In contrast, invasion was significantly reduced in a dose-dependent manner by clinical concentrations of minoxidil and ranolazine. Combining the two drugs produced significant additive effects at concentrations as low as 0.625 μM ranolazine and 2.5 μM minoxidil. Electrophysiologically, acute application of minoxidil shifted VGSC steady-state inactivation to more hyperpolarised potentials and slowed recovery from inactivation, consistent with inhibition of VGSC activation. We concluded (i) that clinically relevant doses of minoxidil and ranolazine individually could inhibit cellular invasiveness dose dependently and (ii) that their combination was additionally effective. Accordingly, ranolazine, minoxidil and their combination may be repurposed as novel anti-metastatic agents.

**Supplementary Information:**

The online version contains supplementary material available at 10.1007/s10585-022-10166-7.

## Introduction

Breast cancer (BCa) has surpassed lung cancer as the most commonly diagnosed cancer in women, with an estimated 2.3 million new cases annually [1]. Of these, the most aggressive and difficult to treat subtype (ca. 20% of cases) is ‘triple-negative breast cancer’ (TNBC) which lacks expression of estrogen, progesterone and human epidermal growth factor HER2 receptors. Hence, biological therapy for this kind of cancer is not readily possible. Lasting response of TNBC to chemotherapy is limited (with recurrence in some 40% of patients with stage I-III disease) resulting in poor prognosis [2, 3]. Patients with TNBC have a three-year survival rate of ca. 44% [4]. The main cause of death from TNBC, as in most cancers, is metastasis following relapse [3]. Consequently, new effective therapies are urgently needed for the clinical management of TNBC.

Ion channels are increasingly being suggested as promising novel targets against cancers [5–9]. In particular, an impressive body of evidence has been presented showing that functional voltage-gated sodium channel (VGSC) expression promotes, may even initiate, the metastatic process [10–12]. Much of this work has been done on the TNBC model MDA-MB-231 cells in vitro and in vivo [11, 13–17]. The predominant VGSC in MDA-MB-231 cells is nNa_v_1.5, the neonatal splice variant of Na_v_1.5 [14, 18]. Under hypoxic conditions, which occur naturally in growing tumours, this channel develops a ‘persistent current’ (I_NaP_) which may be responsible for the elevated levels of sodium detected in BCa cells and tissues including clinically by ^23^Na-MRI [19–22]. Importantly, selective blockage of I_NaP_ with the anti-angina drug ranolazine significantly inhibits invasiveness in vitro and metastasis in vivo [13, 23, 24]. However, ranolazine at higher concentrations can also influence other ionic mechanisms and can have adverse cardiac effects [25].

Combinations of drugs  can produce enhanced effects against cancer whilst maintaining or improving efficacy, especially if the combined drugs can target key pathways in a synergistic manner [26–28]. In a previous study, in an attempt to optimise the effective concentration of ranolazine, we combined it with propranolol, a ‘beta blocker’ and an inhibitor of VGSCs [24]. Although propranolol was also anti-invasive, the combination did not prove additive; in fact, some ‘antagonism’ was apparent [24].

In the present study, we combined ranolazine with minoxidil to test for another possible synergistic effect. Minoxidil is an activator of K_ATP_ channels which are normally opened by falling intracellular ATP/rising ADP levels [e.g. 29, 30]. Thus, these channels are involved broadly in modulating the membrane potential and cell metabolism [30, 31]. K_ATP_ channels are hetero-octameric complexes containing two rings as subunits—an inner ring of inwardly rectifying potassium channel (Kir6.1 or Kir6.2) and an outer ring of sulphonylurea receptor (SUR1 or SUR2) [30–32].

Minoxidil is used by cancer patients to promote hair growth especially after chemotherapy. A bioinformatic study in which genes differentially expressed between cancer vs. normal tissues were ‘mapped’ onto drugs with modes of action associated with those genes suggested that use of minoxidil would raise breast cancer risk [33]! Experimentally, the effects of minoxidil on cancer cells have been studied mainly in regard to ‘growth’ and the available data are mixed. Thus, on human breast, prostate and colon cancer cells minoxidil was originally reported to *promote* proliferative activity [34–36]. Conversely, a K_ATP_ channel blocker, glibenclamide, inhibited proliferation of breast and cervical cancer cells [37, 38]. In contrast, in a more recent study, minoxidil was shown to have *anti-proliferative* and pro-apoptotic effects on ovarian cancer in vitro and in vivo [39]. Possible effects of minoxidil on invasiveness has not previously been studied.

Taking the available information together, the main aims of this study were (1) to test the possible anti-invasive effect of minoxidil on the TNBC cell line, MDA-MB-231 under conditions where proliferation was not affected; (2) to assess whether combination with ranolazine could produce any additive effects; (3) to gain an insight into the concentration dependence of the combination; (4) to make some comparisons between normoxic and hypoxic conditions; and (5) to determine whether minoxidil would affect nNa_v_1.5 activity.

## Materials and methods

### Cell culture

Human MDA-MB-231 and MDA-MB-468 cells were cultured as described previously [14, 40]. In brief, maintenance was in basic Dulbecco’s Modified Eagle Medium (DMEM) (Invitrogen, Paisley, UK). This was supplemented with 4 mmol/L l-glutamine and 5% foetal bovine serum (FBS) (Invitrogen). Cells were maintained routinely at 37 °C, 21% O_2_, 5% CO_2_ and 100% humidity in an incubator (Heraeus, Hanau, Germany). For plating, cells were treated in the incubator for 5–10 min with trypsin–EDTA (Sigma- Aldrich®, Dorset, UK). Cell were ‘pelleted’ by centrifugation for 1 min at 300 g and re-suspended in culture medium. Cell counts were determined using a haemocytometer. Cells were made hypoxic by exposing the cultures to a reduced level of O_2_ (1%) during treatments in a dedicated hypoxia chamber (Micro Galaxy, RS Biotech Laboratory Equipment Ltd, Irvine, UK).

### Pharmacology

Ranolazine was obtained from Sigma-Aldrich (Dorset, UK). Minoxidil was obtained from Alfa Aesar TM (Thermo Fisher Scientific, UK). Four concentrations of ranolazine were used: 0.625, 1.25, 2.5 and 5 μM; three concentrations of minoxidil were used: 2.5, 5 and 50 μM. Stock solutions of ranolazine (2 mM) and minoxidil (31 mM) were prepared by dissolving the drugs in DMEM and 100% dimethyl sulfoxide (DMSO), respectively (Sigma-Aldrich). The stocks were kept frozen at − 20 °C until use. Control solutions for minoxidil and combined treatment contained the final concentration of DMSO. Fresh solutions were made at desired concentrations by dilution in DMEM and warming to 37 °C prior to each experiment. Treatments were either short‐term/acute (electrophysiology) or long‐term/48 h (electrophysiology and functional assays).

### Cell viability and proliferation

Effects of minoxidil, ranolazine and their combinations on cell viability under both in normoxic and hypoxic conditions were determined using the trypan blue dye exclusion assay. Upon completion of a given treatment period, the medium was aspirated and replaced for 10 min with 0.2 mL of 0.4% trypan blue (Sigma-Aldrich) and 0.8 mL of the DMEM medium. The trypan blue solution was then replaced with 1 mL DMEM, and the dishes were counted at × 200 magnification on an inverted microscope (ID 03, Zeiss). The 3-(4,5-dimethylthiazol-2-yl)-2,5-diphenyltetrazolium bromide (MTT) assay was used to quantify ‘cell number’ which was assumed to represent proliferation when cell viability was not affected. In brief, 2 × 10^4^ cells were plated in 24‐well plates and after 48 h of drug treatment, the solution was replaced with 100 µL 5 mg/ml MTT + 400 μL culture medium. After 3 h, the MTT solution was replaced with 500 µL DMSO and 67.5 µL glycine buffer. The plates were then shaken in darkness for 5 min and the resulting formazan absorbance was measured on a plate reader at 570 nm (ELX800 Universal Microplate Reader; Bio‐Tek Instruments, UK). Cell numbers were deduced from a standard curve showing a linear relationship between cell number and absorbance (Supplementary Data).

### Polymerase chain reaction

Steps for polymerase chain reactions (PCRs) were as described before [14]. Briefly, quantitative real-time PCRs were carried out utilising SYBR Green technology (Qiagen) and a AriaMx Real-time PCR system (Agilent Technologies, Didcot, UK). Triplicate reactions on each sample were carried out simultaneously for target and reference genes. For the latter, PUM1 was adopted [41]. Control PCRs were carried out routinely by including non-target (-RT) reactions and monitoring melting curves. The primer pairs used for Kir6.1, 6.2 and SUR1/2A/2B were as described previously [37]. Agilent AriaMx software v1.5 was used to determine cycle threshold (Ct) values for each sample. The mRNA levels were quantified using the comparative 2[exp-ΔΔC(t)] method [42].

### Matrigel invasion

The invasion assay was performed as described previously [14]. The total treatment time with drugs was 48 h with a pre-treatment time of 28–36 h. Transwell filters with 8 μm pores were placed in 24-well companion plates and coated with 50 µL of 1.25 mg/mL Matrigel® (Becton Dickinson). The coated filters were left in the incubator overnight. Prior to cell plating, the inserts were rehydrated with FBS-free DMEM. The pre-treated cells were trypsinized, resuspended in the treatment solution supplemented with 1% FBS and 2 × 10^4^ cells were seeded into the upper chamber of the inserts. A chemotactic gradient was created by adding 300 μL of treatment solution supplemented with 1% FBS to the upper chamber and an equal volume with 5% FBS to the lower chamber. Following completion of the invasion period under either hypoxic or normoxic conditions, the solutions in both chambers were aspirated and the upper part of the insert was swabbed to remove the non-invaded cells and Matrigel. Invaded cells were fixed for 15 min with 300 μL of ice-cold 100% methanol and stained with 300μL of 0.5 g/mL crystal violet diluted in 25% methanol. After washing with distilled water, 20 randomly chosen fields of view were evaluated on an inverted microscope at × 400 magnification (Carl Zeiss, Hertfordshire, UK).

### Electrophysiology

Whole-cell patch clamp recordings were performed on cells under superfusion with mammalian physiological saline (MPS). Details of the whole-cell recordings have been described previously [14, 43–45]. In brief, MPS contained (in mM): 144 NaCl, 5.4 KCl, 1 MgCl_2_, 2.5 CaCl_2_, 5 HEPES and 5.6 d-glucose (adjusted to pH 7.3 with NaOH). Patch pipettes (tip resistances, ~ 5 MΩ) were filled with a solution designed to block the outward K^+^ currents (in mM): NaCl 5, CsCl 145, MgCl_2_ 2, CaCl_2_ 1, HEPES 10 and EGTA 11, adjusted to pH 7.4 with 1 M CsOH. The estimated intracellular free Ca^2+^ concentration was ~ 15 nM [46]. A holding potential of − 100 mV was applied. Standard voltage-clamp protocols were used to study the electrophysiological properties of the VGSC currents. Mainly the following characteristics were studied: peak current (and its density); current–voltage relationship (current normalized to peak): steady-state inactivation (“availability”) = test current (I)/maximum current (I_max_); recovery from inactivation = test current (I_t_)/control current (I_c_). For accurate determination of the acute effect of minoxidil on peak current block, only currents larger than 200 pA were used. Further details of the voltage-clamp protocols, data analysis and curve fitting were published earlier [47].

### Data analysis

A minimum of three biological repeats, each consisting of at least 3 technical repeats were performed.  For invasion assays, a minimum of three biological repeats, each biological repeat consisting of 2 inserts were undertaken. In order to calculate the percentage reduction in invasiveness, the number of invaded cells in each chosen field of view was normalized with respect to the highest value for each insert. Then the percentage reduction was calculated by dividing the normalized value for the treatment insert by the normalized value for the control insert. Normality of data was checked with the Shapiro–Wilk test. Parametric data were analysed with a Student’s *t*-test and displayed via bar graph (showing means ± standard errors of the mean). Non-parametric data was analysed using a Mann–Whitney *U*-test and was displayed via box plots (showing medians, interquartile range; 5% and 95% confidence intervals and outliers). Significant results are indicated as *(P < 0.05), **(P < 0.01) or ***(P < 0.001).

## Results

**Fig. 1:**
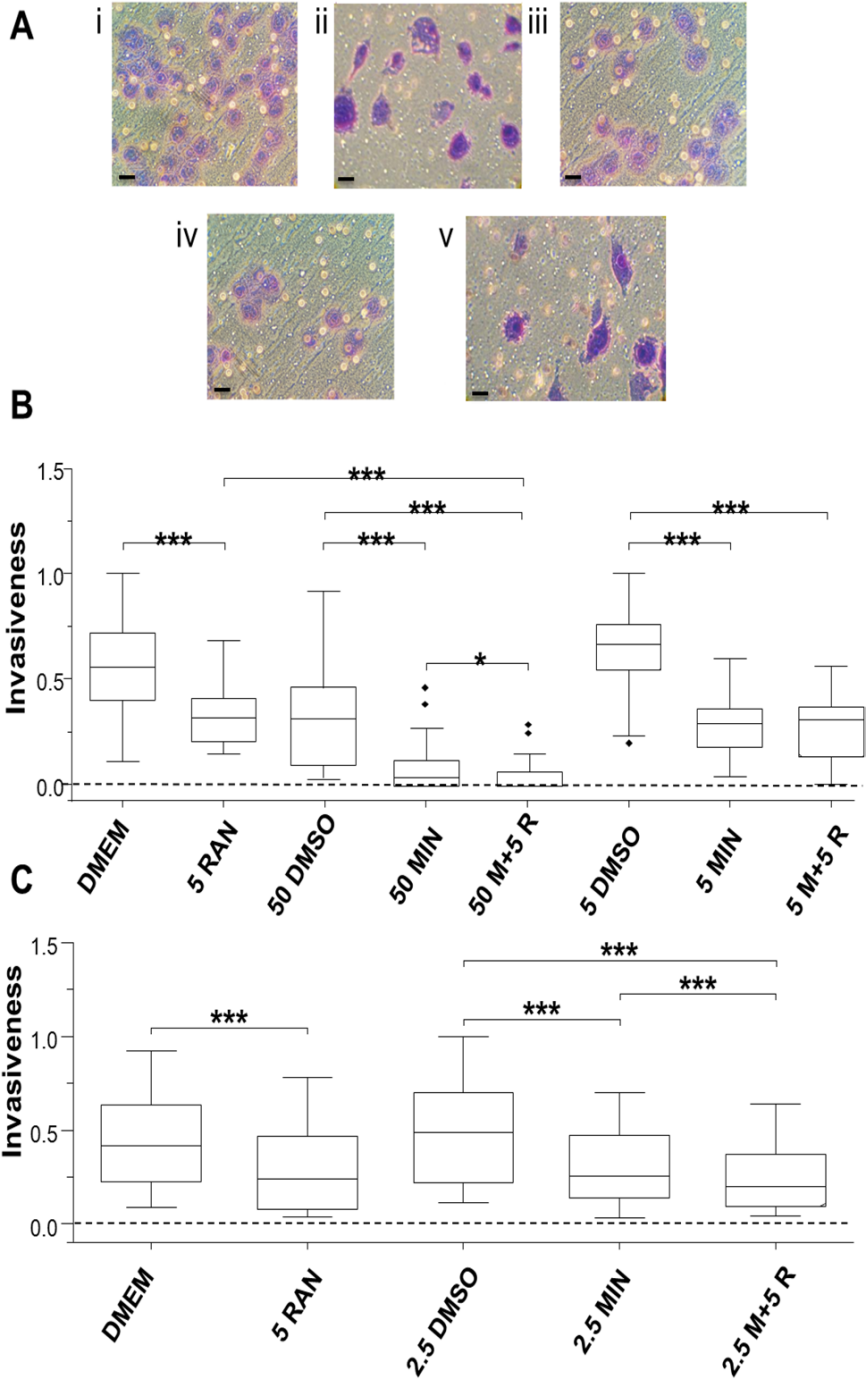
Effects of minoxidil (MIN), ranolazine (RAN) and their combination on invasiveness of MDA-MB-231 cells under hypoxia. **A** Representative images showing stained MDA-MB-231 cells having invaded under (i) DMEM control; (ii) 5 µM RAN; (iii) DMSO control; (iv) 50 µM MIN and (v) combination drugs conditions. The 15 µm scale bar is shown on the bottom left of each image. Box plots showing the effects of 50 and 5 µM MIN, 5 µM RAN and their combination (n = 4) (**B**); and 2.5 µM MIN and 5 µM RAN and their combination (n = 3) (**C**). The Y-axis represents invasion normalized to the largest number of invaded cells viewed per insert. The box plots are presented as medians, interquartile range; 5% and 95% confidence intervals and outliers. Statistical significance is indicated as defined in Data Analysis

**Fig. 2:**
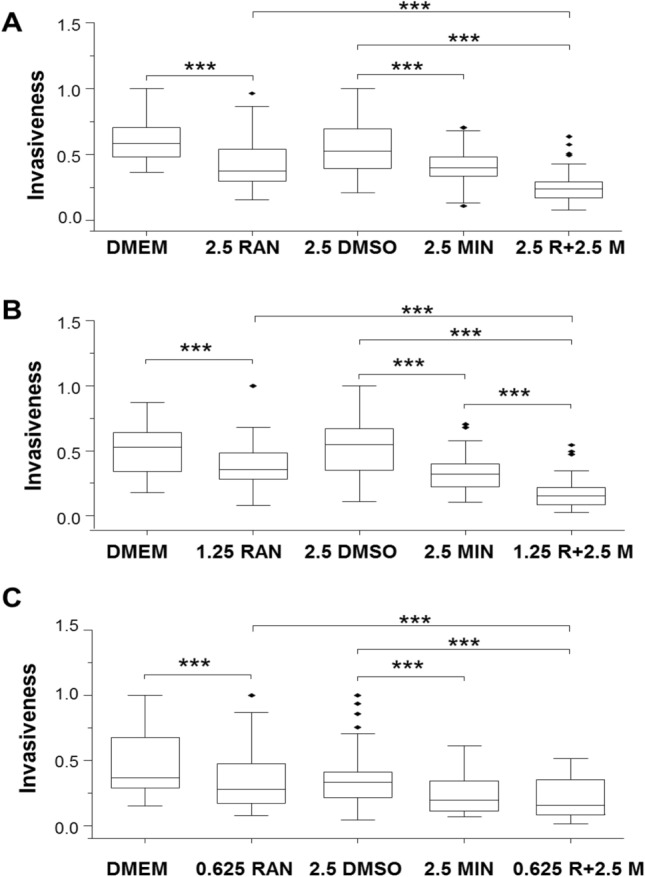
Effects of lowering the dose of ranolazine (RAN) at fixed dose of minoxidil (MIN), and their combination on invasiveness of MDA-MB-231 cells under hypoxia. Box plots showing the effects of the following treatments: **A** 2.5 µM MIN, 2.5 µM RAN and their combination (n = 3). **B** 2.5 µM MIN, 1.25 µM RAN and their combination (n = 6). **C** 2.5 µM MIN, 0.625 µM RAN and their combination (n = 4). The Y-axis represents invasion normalized to the largest number of invaded cells viewed per insert. The box plots are presented as medians, interquartile range; 5% and 95% confidence intervals and outliers. Statistical significance is shown as ***P < 0.001

**Fig. 3:**
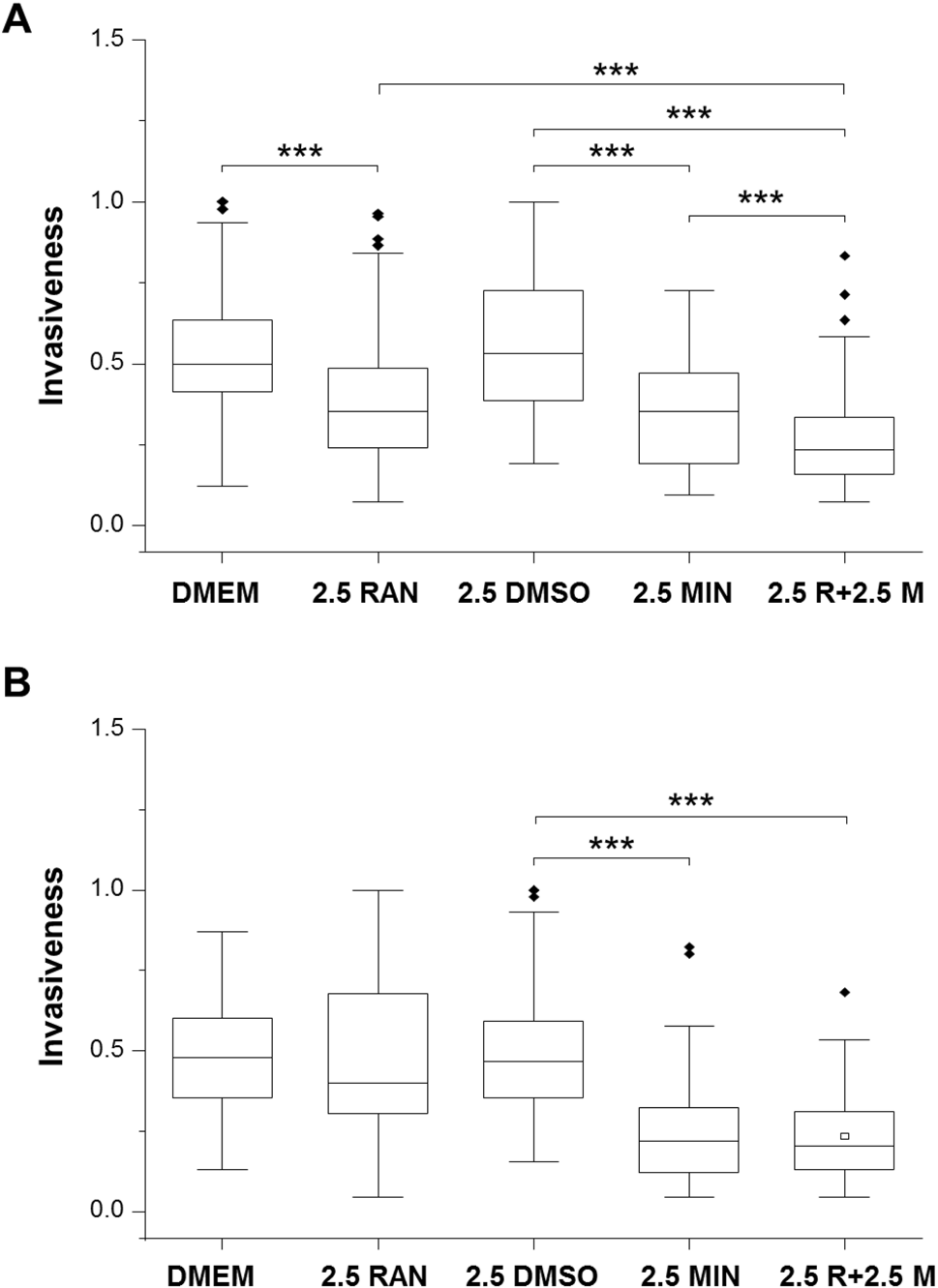
Effects of minoxidil (MIN), ranolazine (RAN) and their combination on invasiveness of MDA-MB-231 cells: Comparison of hypoxic and normoxic conditions. Box plots showing the effects of 2.5 µM MIN, 2.5 µM RAN and combination treatments on cell invasiveness under **A** hypoxia and **B** normoxia (n = 3 for both). The Y-axis represents invasion normalized to the largest number of invaded cells viewed per insert. The box plots are presented as medians, interquartile range; 5% and 95% confidence intervals and outliers. Statistical significance is shown as; ***P < 0.001

**Fig. 4:**
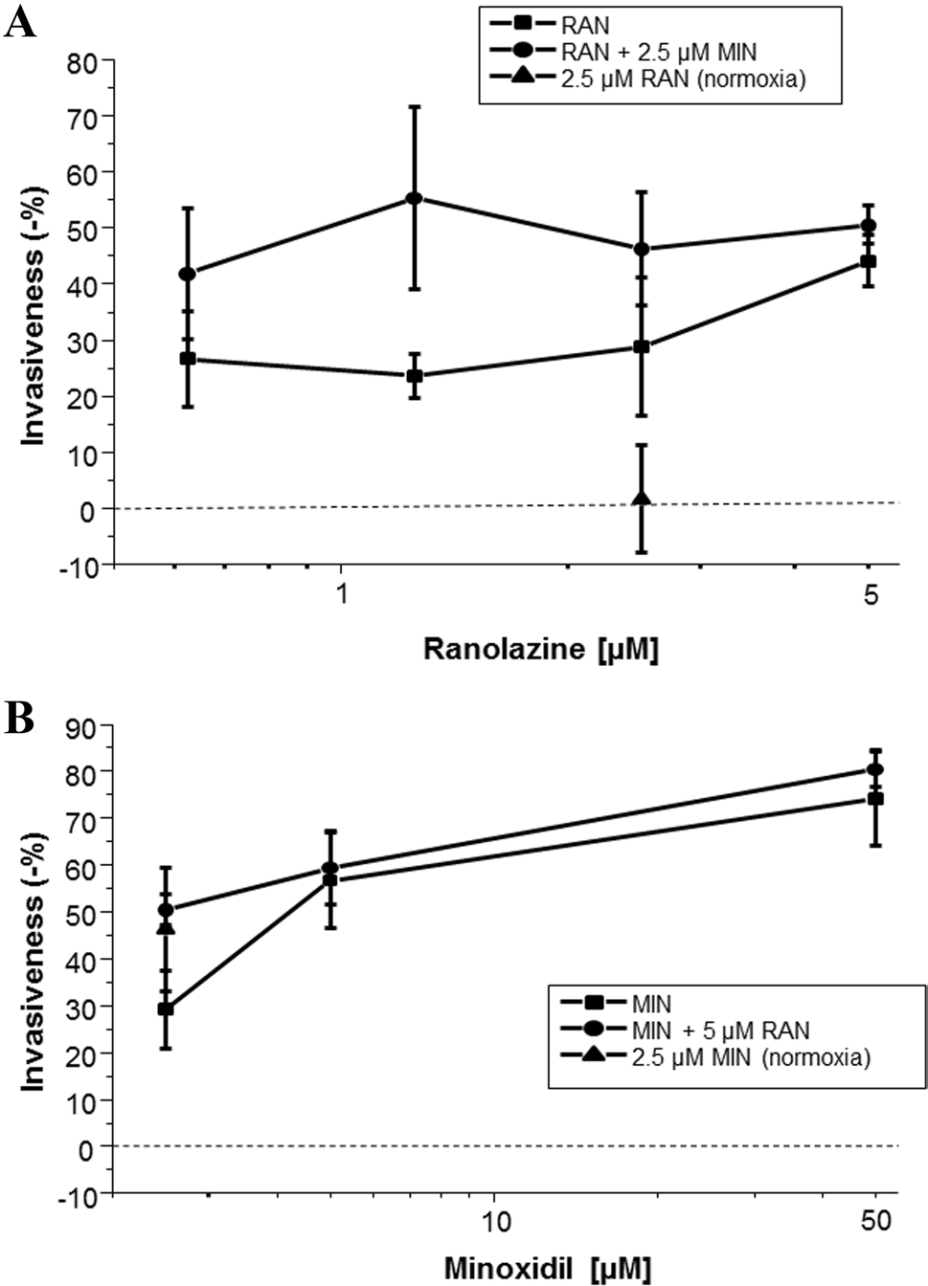
Dose-dependent effects of minoxidil (MIN) and ranolazine (RAN) and their combination on invasion under hypoxia and comparison with normoxia. Graphs showing the effects of **A** 0.625–5 µM RAN and the combination with 2.5 µM MIN and **B** 2.5–50 µM MIN and the combination with 5 µM RAN on reducing cell invasiveness under hypoxia. Also shown are effects of **A** 2.5 µM RAN and **B** 2.5 µM MIN on cell invasiveness under normoxia. The Y-axis represents the percentage reduction in invasion. The data are presented as means ± standard error of the means. Data are from n = 6–14 experimental repeats

**Fig. 5:**
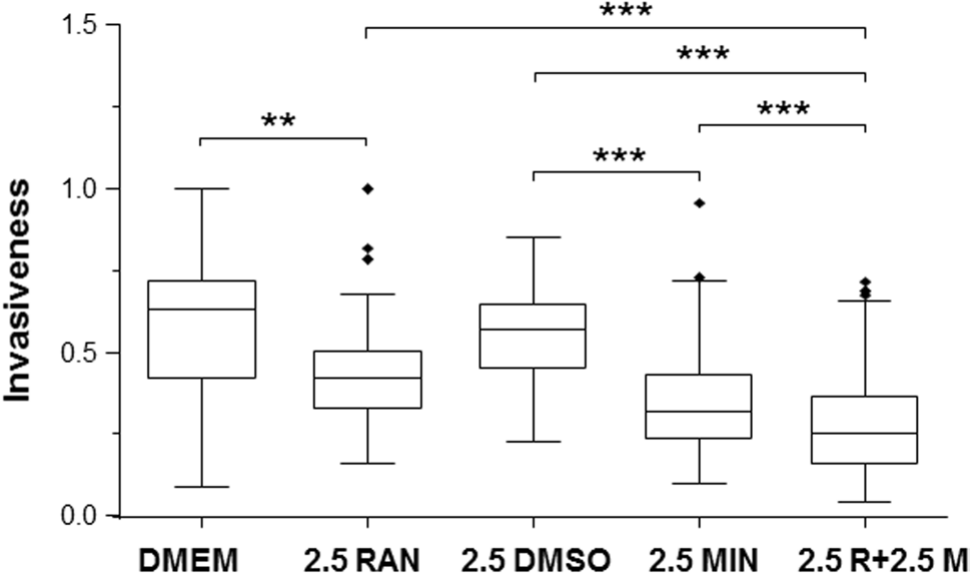
Effects of minoxidil (MIN), ranolazine (RAN) and their combination on invasion of MDA-MB-468 cells. Box plots showing the effects of 2.5 µM MIN, 2.5 µM RAN and combination treatments on cell invasiveness of the MDA-MB-468 cell line under hypoxia (n = 4). The Y-axis represents invasion normalized to the largest number of invaded cells viewed per insert. The box plots are presented as medians, interquartile range; 5% and 95% confidence intervals and outliers. Statistical significance is shown as; **P < 0.01 and ***P < 0.001

Effects of varying concentrations of minoxidil (MIN), ranolazine (RAN) and their combinations on invasiveness of MDA-MB-231 cells under normoxic and hypoxic conditions are shown in Figs. 1, 2 and 3; dose-dependence of the data are compiled in Fig. 4. A second invasive BCa cell line (MDA-MB-468) was used for comparison (Fig. 5). All experiments were carried out in integral sets, including controls, of the three drug treatments at given concentrations.

### Initial control experiments

Quantitative PCRs on MDA-MB-231 and MDA-MB-468 cells confirmed expression of K_ir_6.1, 6.2 and SUR1/2A/2B subunits (Supplementary Fig. 1). There was considerable variability but no difference in the expression levels for any of the subunits between the two cell lines. The highest concentrations of 5 μM ranolazine (RAN), 50 μM minoxidil (MIN) and their combinations, as used in the treatments, had no effect on cell viability or proliferation under normoxic or hypoxic conditions over 48 h (Supplementary Figs. 2 and 3).

### Effects of fixed dose of ranolazine (5 μM), variable doses of minoxidil (2.5–50 μM) and their combinations on Matrigel invasiveness of MDA-MB-231 cells under hypoxia

MIN (50 and 5 μM) reduced median values of invasion by 89% and 56%, respectively (P < 0.001 for both; Fig. 1A). RAN (5 μM) produced a reduction of 42% (P < 0.001; Fig. 1A). Combination of the two drugs was also inhibitory. Thus, 50 μM MIN + 5 μM RAN reduced invasiveness by 100% and this was significantly greater than for both MIN and RAN when applied alone (P < 0.05 and P < 0.001, respectively). Reducing the concentration of MIN in the combination (i.e. 5 μM MIN + 5 μM RAN) still reduced invasiveness, by 54% (P < 0.001; Fig. 1A). This was the same as the effects of the individual treatments. In the next experiment, the concentration of MIN was lowered further (Fig. 1B). Thus, 2.5 μM MIN, 5 μM RAN and their combination suppressed invasiveness by 48, 43 and 58%, respectively (P < 0.001 for all). The effect of the combination was the same as RAN alone, but significantly greater than MIN alone (P < 0.001).

It was concluded (i) that MIN could suppress invasiveness at concentrations as low as 2.5 μM; (ii) that 5 μM RAN inhibited invasiveness; and (iii) that the effectiveness of RAN increased significantly by combining it with 50 (but not 5 or 2.5) μM MIN.

### Effects of lowering the dose of ranolazine at fixed dose of minoxidil, and their combination on invasiveness of MDA-MB-231 cells under hypoxia

Effects of lowering the concentration of RAN and combining these with a fixed concentration of MIN (2.5 μM) were tested. First, 2.5 μM RAN reduced invasion by 36%, 2.5 μM MIN reduced it by 24%, and the combination caused a significant 55% reduction (P < 0.001 for all; Fig. 2A). The effect of the combination was significantly greater than the effect of RAN (but not MIN) alone (Fig. 2A; P < 0.001). Second, 1.25 μM RAN was still effective, reducing invasion by 32%; as before, 2.5 μM MIN significantly reduced invasion, by 41%; their combination caused a significant 71% reduction (P < 0.001 for all; Fig. 2B). The effect of the combination was significantly greater than both RAN and MIN alone (P < 0.001; Fig. 2B). Lastly, 0.625 μM RAN significantly reduced invasion by 24% whilst 2.5 μM MIN significantly reduced invasion by 42%; their combination caused a significant 53% reduction (P < 0.001 for all; Fig. 2C). The effect of the combination treatment was significantly greater than RAN (but not MIN) alone (P < 0.001; Fig. 2C).

It was concluded (i) RAN suppressed invasiveness at concentrations as low as 0.625 μM and (ii) that the effectiveness of 0.625–5 μM RAN was increased significantly by combination with 2.5 μM MIN.

### Comparison of hypoxic and normoxic conditions and an overview of drug dose-dependence

Next, effects of the drug combination were investigated comparatively under normoxia and hypoxia. 2.5 μM MIN significantly reduced invasion by 53% under normoxia cf. 49% under hypoxia (Fig. 3A, B). Both effects were significant relative to their controls (P < 0.001 for both) but were not different to each other (P = 0.49). RAN (2.5 μM) significantly reduced invasion by 30% under hypoxia (P < 0.001; Fig. 3A) but had no effect under normoxia (Fig. 3B). Combination treatment caused 56% reduction under hypoxia and 57% reduction under normoxia (P < 0.001 for both; Fig. 3A, B). Relative to both individual treatments, the effect of the combination was significantly greater under hypoxia (P < 0.001 for both; Fig. 3A). No such enhancement was apparent under normoxia (Fig. 3B).

The data obtained from the repeated sets of experiments, involving effects of different concentrations of RAN and MIN under hypoxia, allowed the compilation of dose-dependence relationships (Fig. 4). At 0.625 μM, RAN was significantly effective in inhibiting invasion by 27 ± 8% and this rose to 44 ± 5% for 5 μM, the highest concentration tested (Fig. 4A). The inhibitory effect of MIN was also dose dependent: 29 ± 8% for 2.5 μM, rising to 79 ± 10% for 50 μM (Fig. 4B). Importantly, at a test concentration of 2.5 μM, the inhibitory effect of RAN was significantly greater under hypoxia vs. normoxia (P < 0.05; Fig. 4A). In contrast, no such difference was observed for 2.5 μM MIN (P = 0.21; Fig. 4B).

It was concluded (i) that the effectiveness of both RAN and MIN in inhibiting invasion under hypoxia was dose-dependent; (ii) that RAN (but not MIN) was not effective under normoxia; and (iii) that the effectiveness 2.5 μM RAN in reducing invasion was increased significantly by combining it with 2.5 μM MIN but this occurred only under hypoxia.

### MDA-MB-468 cells

The effects of MIN, RAN and their combination were also tested, under hypoxia, on an additional TNBC cell line (MDA-MB-468) also known to express functional VGSC activity [40]. 2.5 μM MIN significantly reduced invasion by 44% (P < 0.001; Fig. 5), whilst 2.5 μM RAN significantly reduced it by 33% (P < 0.01; Fig. 5). The combination treatment caused a significant 55% reduction in invaded cells (P < 0.001); this effect was significantly greater when compared to both RAN and MIN alone (P < 0.001; Fig. 5).

It was concluded for the hypoxic condition tested (i) that both RAN and MIN could also reduce MDA-MB-468 cell invasion and (ii) that the inhibitory effect on invasion was significantly greater for the combination of the two drugs.

### Effects of minoxidil on nNa_v_1.5 activity

**Fig. 6:**
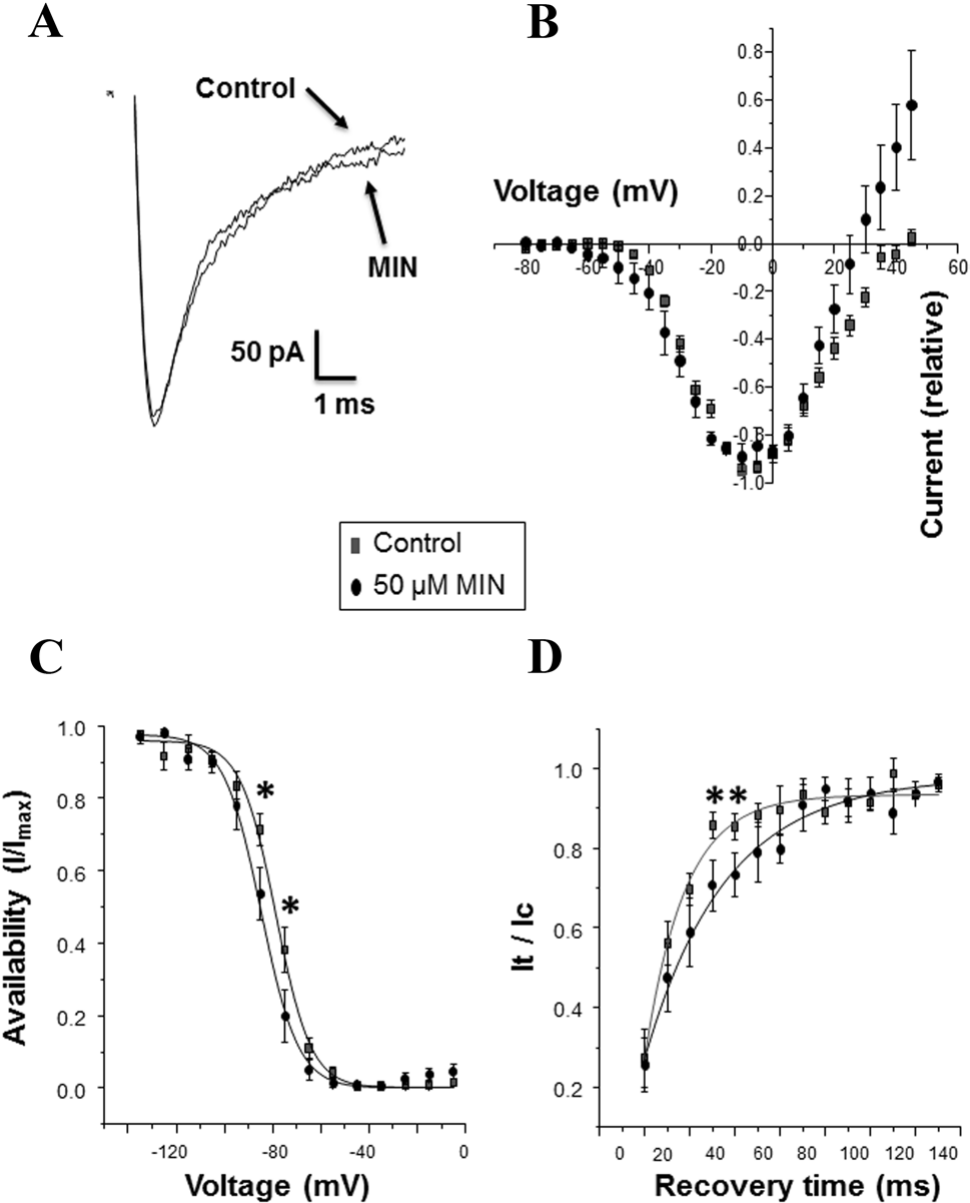
Effect of an acute application of minoxidil (MIN) on nNa_v_1.5 activity in MDA-MB-231 cells. **A** Effect of 50 µM MIN on the peak current. **B** Current–voltage relationship for control conditions and 50 µM MIN (n = 8 and n = 6, respectively). **C** Steady-state inactivation for control cells and 50 µM MIN (n = 7 and n = 5, respectively). **D** Recovery from inactivation for control cells and 50 µM MIN (n = 7 and n = 5, respectively). Key in part (**B**) is relevant to parts (**C**, **D**) of the Figure. The data are presented as means ± standard errors of the mean. Statistical significance for individual data points are shown as; *P < 0.05

(n)Na_v_1.5 activity has previously been shown for MBA-MD-231 cells to be the main driver of invasiveness in vitro and metastasis in vivo [13–15, 18]. We therefore questioned whether MIN would affect the activity of nNa_v_1.5 in MDA-MB-231 cells. Effects of ‘short-term’ (acute) and ‘long-term’ (48 h incubation) treatments were investigated. Acute application of 50 μM MIN had no effect on the peak current (− 2.0 ± 2.1% change; P = 0.98; n = 8 cells) or the current–voltage relationship (Fig. 6A, B). On the other hand, steady-state inactivation was shifted to more hyperpolarised potentials and the recovery from inactivation was slowed, both significantly (P < 0.05; n = 5–7 cells) (Fig. 6C, D). Following 48 h treatment with 50 μM MIN under hypoxia, there was no change in peak current density in comparison to the control: 6.6 ± 1.3 vs. 9.9 ± 2.0 pA/pF (P = 0.19; n = 7–10 cells). Also, there was no change in (i) the proportion of cells expressing functional channel: 88 vs. 83% (P = 1; n = 8–12 cells); (ii) current–voltage relationship; (iii) steady-state inactivation; and (iv) recovery from inactivation (not shown). A similar lack of effect on any VGSC characteristics was also observed for a 48 h treatment with 50 μM MIN under normoxia (not shown).

It was concluded that acute application of MIN could inhibit VGSC (nNa_v_1.5) activity characteristics but, once removed, no long-term change was apparent.

## Discussion

The main results obtained using the MDA-MB-231 cell line model were as follows: (1) At the highest concentrations used, cell viability and proliferation were not affected by MIN (50 μM), RAN (5 μM) or their combination under hypoxia or normoxia. (2) Matrigel invasion was significantly reduced in a dose-dependent manner by MIN under both normoxia and hypoxia; RAN was effective only under hypoxia. The drug combination was also more effective under hypoxia. The data obtained from the different experimental sets were highly consistent. (3) The anti-invasive effect of RAN under hypoxia was apparent at concentrations as low as 0.625 μM and this effect could be increased by combination with MIN. (4) The invasiveness of the MDA-MB-468 cell line was affected similarly by MIN, RAN and their combination under hypoxia. (5) MIN had no apparent long-term effect on electrophysiological characteristics of the VGSC, but acute application shifted steady-state inactivation to more hyperpolarised potentials and slowed recovery from inactivation.

The MDA-MB-231 cells were shown previously to express the K_ATP_ channel subunits K_ir_6.1/6.2 and SUR2B [37]. Here, we have found both MDA-MB-231 and MDA-MB-468 cells to also express SUR1/2A. The reason(s) for these slight differences found in expression between the two studies is, at present, unclear. However, SUR1/2 have previously been shown to be expressed at protein level in MDA-MB-231 cells [48]. Cervical cancer cell lines were found to express K_ir_6.2 and SUR2 subunits at mRNA and/or protein level [38]. The primary mode of action of MIN is activation of K_ATP_ channels with some secondary effects including changes in the cytoplasmic free Ca^2+^ concentration [49–52]. The latter has been reported in a variety of cell types including human breast and prostate cancer cells [53].

Treatment with RAN (5 μM) had no effect on cell viability and proliferation which is in agreement with the currently available in vitro and in vivo data, including previous work on the MDA-MB-231 cells [13, 23, 24]. MIN (50 μM) treatments also showed no effect on cell viability and proliferation. The latter contrasts with an earlier study on MDA-MB-231 cells by Núñez et al. reporting that 5 μM MIN caused a significant increase in cell proliferation [37]. This discrepancy could be due to a number of experimental differences between our study vs. Núñez et al. including, respectively (i) FBS concentration of 5 vs. 10%, which could significantly affect channel expression [e.g. 54; (ii) treatment period of 48 h vs. 10 days, which could lead to differential ‘knock-on’ effects; and (iii) possible difference in the subunit composition of the target K_ATP_ channel protein [39]. Interestingly, MIN was found to reduce ovarian cancer cell proliferation in vitro and primary tumour growth in vivo [39]. However, the K_ATP_ channel blocker, glibenclamide, also inhibited proliferation in breast cancer cells [37] and cervical cancer cells [38]. Further work is required to elucidate these issues. Here, nevertheless, invasiveness was studied under conditions where there was no change in proliferative activity.

Treatments with MIN reduced invasiveness dose-dependently and significantly under both hypoxia and normoxia. There is limited information on possible effects of MIN on cellular invasion. In a hydrogel model of mouse sarcoma, MIN was shown to reduce cell migration and matrix remodelling under hypoxia [55]. In non-cancer smooth muscle cells also, MIN inhibited invasion across a type I collagen membrane [56]. Finally, in an orthotopic assay, the metastatic ability of MDA-MB-231 cells co-injected with adipose tissue was suppressed by MIN [57].

Complete inhibition of the VGSC current by tetrodotoxin (30 μM) has been shown consistently to result in only 35–50% reduction in invasiveness of MDA-MB-231 cells [58, 59]. The noticeably greater effect of MIN  (89% reduction) would suggest (i) enhancement of VGSC activity, as discussed below, and/or (ii) some non-VGSC mechanism(s) also being involved in controlling the cellular invasiveness. As noted above, the latter could involve changes in the level of intracellular Ca^2+^ which could also influence the invasiveness [60].

RAN  (5 μM) reduced invasion in vitro and metastasis in vivo [13, 23, 24]. The fact that the in vitro effect here was seen only under hypoxic conditions is consistent with the following. First, RAN selectively inhibits the persistent component (I_NaP_) of the VGSC/nNa_v_1.5 current [25, 61]. Second, I_NaP_ is promoted by hypoxia [62, 63]. In addition, Guzel et al. have shown for colon cancer that the pro-metastatic role of VGSC/nNa_v_1.5 under hypoxia is likely to be mediated by I_NaP_ [64]. Importantly, the present study has also shown that the effect of RAN is dose-dependent and can still significantly reduce invasion at concentrations as low as 0.625 μM, even below the the estimated anti-anginal therapeutic range of 2–8 µM [65].

RAN and MIN combinations were more effective than either drug at most concentrations tested under hypoxia and generally showed good dose-dependence. This is most likely to be due to RAN and MIN having different but synergistic modes of action—inhibition of I_NaP_ and opening of K_ATP_ channels, respectively [25, 30]. However, it should be noted that effects of combining two drugs with different modes of action can be notoriously unpredictable. For example, in a previous study, we combined ranolazine with propranolol, a ‘beta blocker’ and an inhibitor of VGSCs. Although propranolol was also anti-invasive, the combination did not prove additive; in fact, some ‘antagonism’ was apparent [24]. Overall, however, our results with RAN and MIN would agree broadly with the ‘Celex’ hypothesis of metastasis, i.e. it is driven by concurrent upregulation of VGSC and downregulation of K^+^ channel activity [10]. Thus, MIN application would lead to the opening of K_ATP_ channels, efflux of K^+^ and membrane hyperpolarization [e.g. 66, 67, 68]. This would reduce the probability of VGSC activation and thus inhibit the VGSC-dependent invasiveness. Activating another K^+^ (Kv11.1) channel also suppressed BCa  metastasis in vivo but the possible role of VGSC was not studied [69]. Similarly, BCa cell migration was reduced by up-regulation two-pore domain K^+^ channels [70]. Finally, the reverse was also demonstrated recently whereby blocking K^+^ channel activity led to *increased* invasiveness again of strongly metastatic prostate (rat) and breast (human) cancer cells with some evidence for VGSC involvement [71]. It is worth noting that, at present, there is little direct data linking metastasis, metabolism and K_ATP_ channel activity in cancer cells. It is well known that cancer cells have higher ATP production levels than normal cells [72]. Under such circumstances, K_ATP_ channels would tend to remain closed which could be why MIN proved so efficient in inhibiting invasiveness. In future experiments, it will be interesting also to determine the effects of MIN, RAN and their combination on K^+^ channels (outward currents) and on action potential activity known to occur spontaneously in these cells [73].

Electrophysiologically, acute application of 50 μM MIN slowed the recovery time and shifted the steady-state inactivation to more hyperpolarised potentials, implying that the VGSC would be less likely to be active. This could contribute to the observed inhibitory effects of MIN on invasiveness. In comparison, long-term (48 h) treatment with 50 μM MIN showed no effect on any of the VGSC characteristic under both hypoxia and normoxia. Thus, presence of MIN would seem to be necessary for its impact on VGSC activity and, in turn, invasiveness. This is consistent with the proposed action of MIN on the membrane potential which, indeed, would be rapidly reversible. Interestingly, VGSC (Na_v_1.5) and K_ATP_ channels were also shown recently to be functionally coupled through AnkyrinG in both heterologous expression systems and cardiac myocytes [74]. Functionally also, in neurons, intracellular sodium accumulation could modulate K_ATP_ channel opening [75]. Further study of VGSC (Na_v_1.5) and K_ATP_ channel interaction would require silencing of the K_ATP_ channel. Interestingly, also, MIN treatment appeared to cause a notable increase in the outward current around the region of the reversal potential (Fig. 6B). Although not studied in detail, this could simply be due to increased efflux of K^+^ through the presumed enhancement of K_ATP_ channel activity.

## Conclusions and future perspective

This study was motivated by the dual possibility of (i) repurposing neuroactive drugs as anti-metastatic agents and (ii) determining possible synergistic effect of such drug combinations [5, 6, 27, 76]. Such a strategy has already been highlighted for BCa  and could lead to novel cancer treatments using drug(s) with well-characterised safety and pharmacokinetic profiles, thereby speeding up the clinical approval process for the benefit of patients [77]. In these respects, combining RAN as ‘backbone’ medication with MIN would seem highly promising and would warrant more detailed  studies including of additional TNBC cell lines. In addition, co-localization of the channels in tissues and in vivo experiments (including xenograft mouse models) could be employed to further establish and strengthen the relevance of using this combination treatment. Minoxidil could also be useful in reducing chemotherapy-induced peripheral neuropathy [78]. Further studies on other K_ATP_ channel openers, such as pinacidil and diazoxide, could enable additional combinations. All these could be extended to other cancers also known to express functional VGSCs [11]. Finally, we should note that repurposing and combination therapies of cancer could be aided by computational modelling [79].

## Supplementary Information

Below is the link to the electronic supplementary material.

Supplementary file1 (PDF 132 kb)

## Data Availability

All data generated or analysed during this study are available on request from the authors.
