# Supplementary material for: Anti-invasive effects of minoxidil on human breast cancer cells: combination with ranolazine
Source: Clin Exp Metastasis. 2022 May 28;39(4):679–89. doi: 10.1007/s10585-022-10166-7 (PMC9338910; doi:10.1007/s10585-022-10166-7)
Supplement: Supplementary file 1 — Supplementary file1 (PDF 132 kb) [file 10585_2022_10166_MOESM1_ESM.pdf]

SUPPLEMENTARY DATA

Anti-invasive effects of minoxidil on human breast cancer cells: Combination with ranolazine

Shiwen Qiu, Scott P Fraser, Wayne Pires and

Mustafa B A Djamgoz

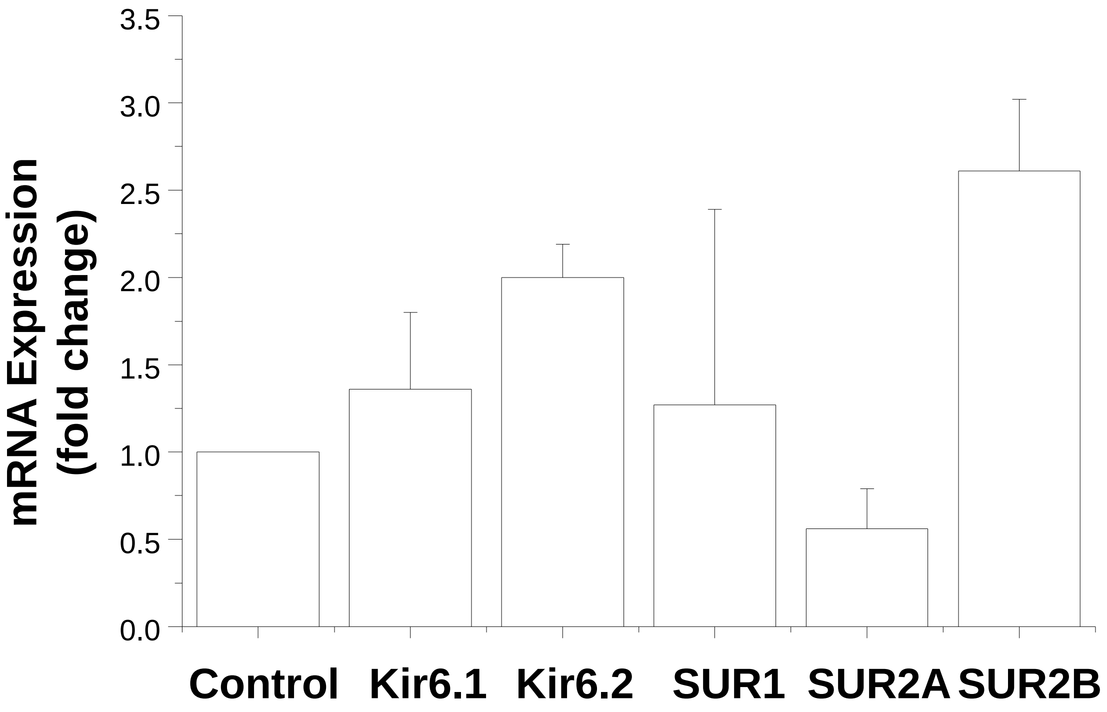

Supplementary Figure 1. Expression of K_{ATP} channel subunit Kir6.1, Kir6.2, SUR1, SUR2A and SUR2B mRNA levels in MDA-MB-231 and MDA-MB-468 cells. Histogram shows the expression levels in MDA-MB-468 cells relative to MDA-MB-231 cells (indicated as Control). Data are shown as means ± standard errors of the mean (n=3).

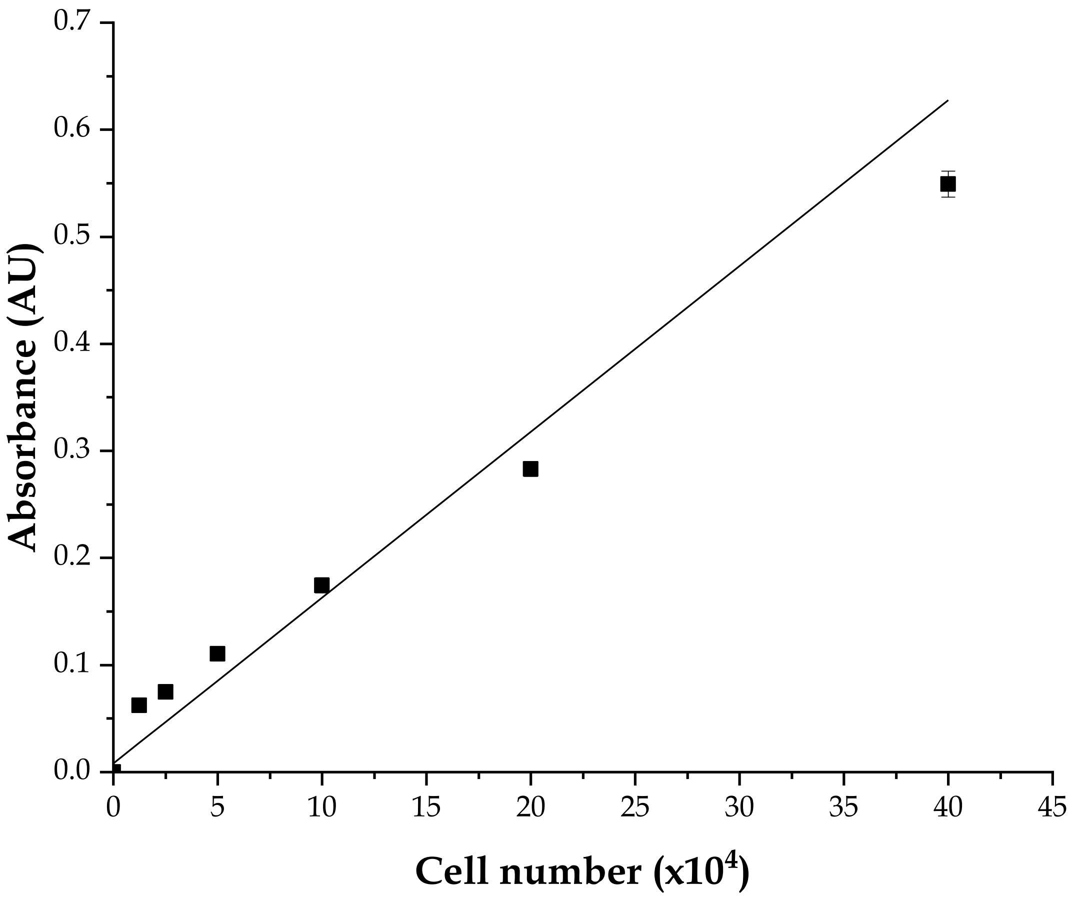

Supplementary Figure 2. Typical calibration graph for the MTT colorimetric assay. Standard curve for the MDA-MB-231 cells showing the linear relationship between absorbance at 570 nm in arbitrary units (AU) and cell number (r = 0.80). Data points indicate means ± SEMs (n=4).

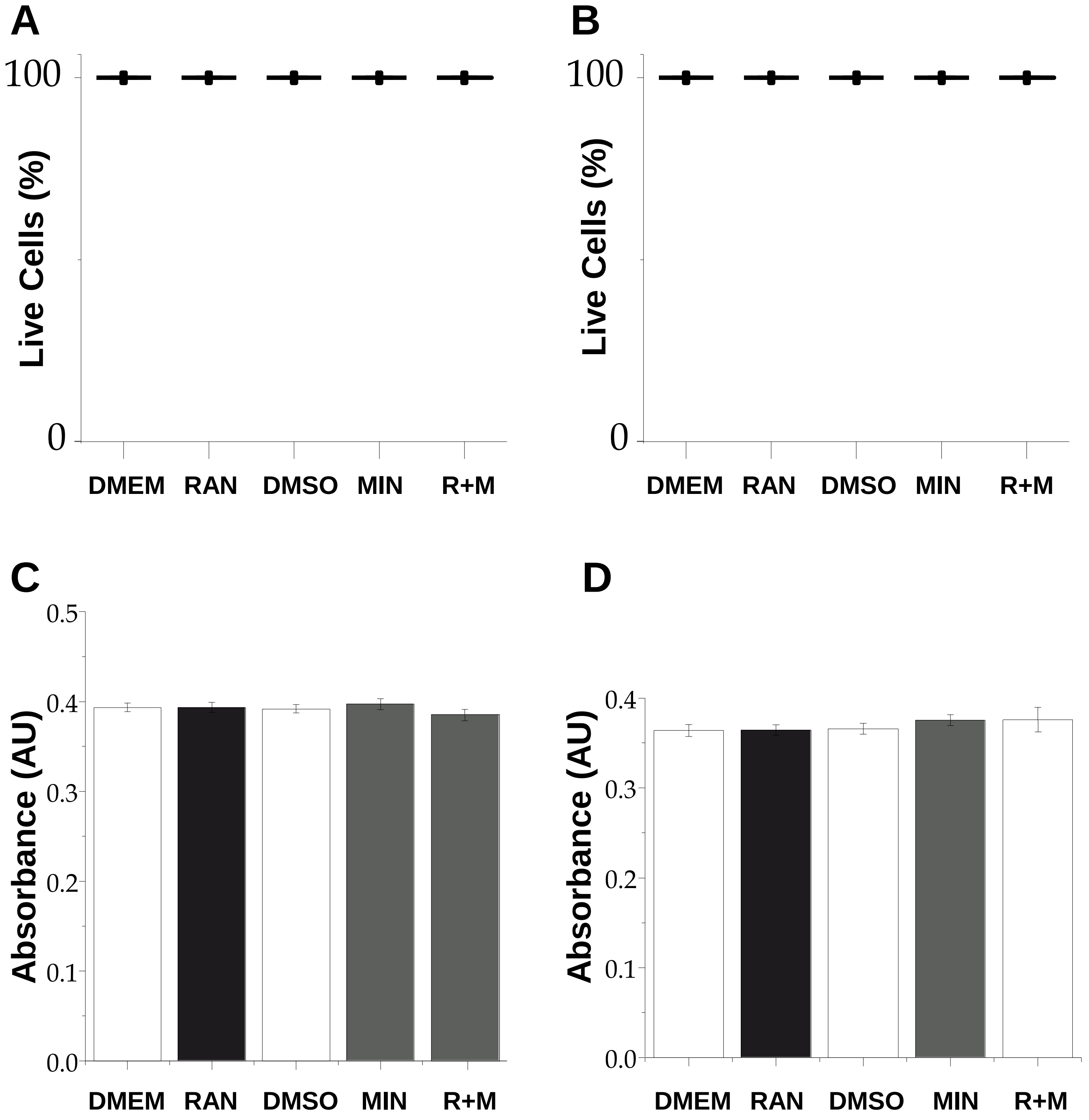

Supplementary Figure 3 Plots showing the effects of ranolazine, (RAN), minoxidil (MIN) and combination treatment (R+M) on cell viability and proliferation of the MDA-MB-231 cells. Cell viability data under normoxia (A) and hypoxia (B) are presented as box plots showing medians, interquartile ranges, 99% and 1% confidence intervals (n=3). Proliferation data under normoxia (C) and hypoxia (D) are presented as histograms showing the mean ± SEM. (n=6) There was no statistical difference for any treatment versus its respective control for both cell viability and proliferation data..
